# The Correlation Between Hepatitis B Virus Precore/Core Mutations and the Progression of Severe Liver Disease

**DOI:** 10.3389/fcimb.2018.00355

**Published:** 2018-10-22

**Authors:** Ahmed A. Al-Qahtani, Mashael R. Al-Anazi, Nyla Nazir, Ayman A. Abdo, Faisal M. Sanai, Waleed K. Al-Hamoudi, Khalid A. Alswat, Hamad I. Al-Ashgar, Mohammed Q. Khan, Ali Albenmousa, Ahmed El-Shamy, Salah K. Alanazi, Damian Dela Cruz, Marie Fe F. Bohol, Mohammed N. Al-Ahdal

**Affiliations:** ^1^Department of Infection and Immunity, Research Center, King Faisal Specialist Hospital and Research Center, Riyadh, Saudi Arabia; ^2^Department of Microbiology and Immunology, Alfaisal University School of Medicine, Riyadh, Saudi Arabia; ^3^Section of Gastroenterology, Department of Medicine, College of Medicine, King Saud University, Riyadh, Saudi Arabia; ^4^Liver Disease Research Center, King Saud University, Riyadh, Saudi Arabia; ^5^Gastroenterology Unit, Department of Medicine, King Abdulaziz Medical City, Jeddah, Saudi Arabia; ^6^Gastroenterology Unit, Department of Medicine, King Faisal Specialist Hospital and Research Center, Riyadh, Saudi Arabia; ^7^Department of Gastroenterology, Prince Sultan Military Medical City, Riyadh, Saudi Arabia; ^8^Department of Pharmaceutical and Biomedical Sciences, California Northstate University, Elk Grove, CA, United States

**Keywords:** HCC, hepatitis, cirrhosis, core gene, mutations, HBV

## Abstract

Viral mutations acquired during the course of chronic hepatitis B virus (HBV) infection are known to be associated with the progression and severity of HBV-related liver disease. This study of HBV-infected Saudi Arabian patients aimed to identify amino acid substitutions within the precore/core (preC/C) region of HBV, and investigate their impact on disease progression toward hepatocellular carcinoma (HCC). Patients were categorized according to the severity of their disease, and were divided into the following groups: inactive HBV carriers, active HBV carriers, liver cirrhosis patients, and HCC patients. Two precore mutations, W28^*^ and G29D, and six core mutations, F24Y, E64D, E77Q, A80I/T/V, L116I, and E180A were significantly associated with the development of cirrhosis and HCC. Six of the seven significant core mutations that were identified in this study were located within immuno-active epitopes; E77Q, A80I/T/V, and L116I were located within B-cell epitopes, and F24Y, E64D, and V91S/T were located within T-cell epitopes. Multivariate risk analysis confirmed that the core mutations A80V and L116I were both independent predictors of HBV-associated liver disease progression. In conclusion, our data show that mutations within the preC/C region, particularly within the immuno-active epitopes, may contribute to the severity of liver disease in patients with chronic hepatitis. Furthermore, we have identified several distinct preC/C mutations within the study population that affect the clinical manifestation and progression of HBV-related disease. The specific identity of HBV mutations that are associated with severe disease varies between different ethnic populations, and so the specific preC/C mutations identified here will be useful for predicting clinical outcomes and identifying the HBV-infected patients within the Saudi population that are at high risk of developing HCC.

## Introduction

Despite improvements in the availability of effective vaccines and antiviral medications, hepatitis B virus (HBV) infection continues to be a significant global health problem. According to the World Health Organization, ~240 million people are chronically infected with HBV, and an estimated 686,000 people each year die from HBV-related complications, including cirrhosis and liver cancer (WHO, 2016). Since implementing a national vaccination program, Saudi Arabia has experienced a significant reduction in the proportion of the population infected with HBV (Ayoola et al., 2003; Madani, 2007), although the prevalence of the HBV surface antigen (HBsAg), indicating active infection, in adults above 40 years of age remains concerning at 3–6% (Abdo et al., 2012). HBV incidence among Saudi inhabitants was reportedly 14.7 per 100,000 in 2013, and 23,236 new cases were recorded between 2009 and 2013 (Algarni, 2014). Saudi Arabia has a large expatriate population, most of whom originate from countries with endemic HBV infection (Bashawri et al., 2004). Several genetic factors, in both the host and the virus, influence susceptibility to HBV infection, virus clearance efficiency, and the maintenance of chronic infection and progression toward the advanced stages of HBV-related liver diseases. Indeed, recent studies have focused on the relationship between HBV strain variants and the clinical severity of liver diseases, particularly hepatocellular carcinoma (HCC) (Kim et al., 2012). HBV has a relaxed circular, partially double-stranded DNA genome, that is approximately 3.2 kb in length and contains 4 overlapping open reading frames (ORFs) encoding the polymerase (P), core (C), surface antigen (S), and X proteins (Zhang et al., 2016). HBV mutations arise primarily from a lack of HBV reverse transcriptase proofreading activity, and from pressure from the host immune system (Desmond et al., 2012). HBV genomic mutations reportedly affect viral replication and disease progression, and can affect therapeutic outcomes (Malik et al., 2012). Indeed, the relationships between many HBV mutations and the clinical severity of resulting diseases have been documented. The pre-S2 mutation F141L and the pre-S1 mutation W182^*^, leading to the premature termination of HBsAg, are reportedly associated with more severe HBV infection (Mun et al., 2011; Lee et al., 2012). Additionally, several studies have reported that point mutations, particularly those in the S gene “a” determinant region, can arise during the natural course of HBV infection, and result in immune escape, active viral replication, and liver disease progression (Mathet et al., 2003; Lada et al., 2006). Mutations within the X gene, including V5M, I127T, K130M, and V131I/L, are risk factors for HCC and may promote the progression of liver degradation (Kim et al., 2008). Similarly, the role of the precore mutation G1896A and the basal core promoter double mutation A1762T/G1764A in the progression of HBV-related liver diseases has been intensively studied (Kim et al., 2016).

The precore/core ORF has two initiation (AUG) codons, which lead to the synthesis of two distinct proteins; initiation from the first codon leads to the synthesis of the precursor for HBV e antigen (HBeAg), a key HBV immune regulatory protein, while initiation from the second codon results in the translation of the nucleocapsid or HBV core antigen (HBcAg). HBcAg is the primary target for the host immune response, particularly cytotoxic T lymphocyte attack. Many reports describe non-synonymous HBcAg mutations that encode variants with altered immune epitopes, which are more able to escape immune surveillance and are more likely to cause severe liver disease (Ehata et al., 1993; Bozkaya et al., 1996; Okumura et al., 1996; Seeger, 2000; Preikschat et al., 2002; Bock et al., 2003; Kim et al., 2011). HBcAg mutations generally occur within the MHC restricted region of HBV, and higher MHC class II mutation rates have been reported in patients with HBV-induced HCC than in patients without HCC (Ferrari et al., 1991; Kim et al., 2012). Furthermore, five HBcAg mutations identified in a Korean population, P5H/L/T, E83D, I97F/L, L100I, and Q182K/Stop, were shown to be significantly enriched in HCC patients compared with patients at earlier disease stages, including those with cirrhosis and chronic hepatitis (Kim et al., 2012). Similarly, a study of an Indian population showed that HBcAg residue 5 mutations P5H/L/T or P5R were found more frequently in HCC patients than in HBV-infected patients at other clinical stages of liver disease (Malik et al., 2012).

The majority of the reported precore mutations are associated with either reduced HBeAg levels or reduced HBV replication in patient sera (Kim et al., 2016). One of the most common precore mutations reported thus far is a G1896A, which prevents HBeAg production through the replacement of a tryptophan residue at amino acid position 28 with a premature stop codon (Tong et al., 2005). This premature stop codon was detected in more than 50% of individuals with chronic hepatitis B in Asia and the Mediterranean area (Tong et al., 2005), and this location has previously been suggested to be a mutational hotspot, leading to the inhibition of HBeAg production and the progression of liver disease (Carman et al., 1989). Furthermore, precore mutations at position A1899 were reportedly associated with cirrhosis and HCC development in HBV-infected patients in a Chinese population (Zheng et al., 2011).

Numerous studies have described a relationship between the frequency of mutation in the precore/core (preC/C) region and the progression of liver disease in HBV-infected patients. However, the association between these mutations and the clinical severity of HBV-induced disease appears to vary substantially between studies and populations. This apparent inconsistency can be attributed to various factors, including HBV genotype, patient ethnicity, host immune competence, and co-infection with other viruses, such as HIV or HCV (Kim et al., 2016). The aim of the present study was to determine the prevalence of preC/C mutations in Saudi Arabian HBV-infected patients at various clinical stages, and to elucidate the relationship between HBV mutations and HBV-related liver disease progression.

## Materials and methods

### Subjects

For this study, we recruited 553 HBV-infected patients from three hospitals in Riyadh, Saudi Arabia: King Faisal Specialist Hospital and Research Center, King Khalid University Hospital, and the Prince Sultan Military Medical City. All study participants provided written informed consent prior to enrollment. The study protocol was approved by the institutional review boards of all of the centers and conformed to the 1975 Declaration of Helsinki. Chronic HBV infection was diagnosed by the consistent detection of HBsAg in patient sera over a 6-month period. Patients were assigned to the following groups based on disease severity: Case I-inactive HBV carriers (IC), including patients testing positive for HBsAg and negative for HBeAg, with persistently normal serum alanine aminotransferase (ALT) levels (40 U/L); Case II-active HBV carriers (AC), including patients testing positive for HBsAg, with elevated serum ALT levels and no evidence of liver complications; Case III-patients with HBV infection and liver cirrhosis (LC), confirmed by liver biopsy, and clinical, biochemical, or radiological evidence of cirrhosis; Case IV-patients with HCC diagnosed by computed tomography and/or magnetic resonance imaging of the liver, using previously published guidelines for the diagnosis and management of HCC (Abdo et al., 2006).

### Nucleic acid extraction from serum

Viral DNA was extracted from 200 μL of each serum sample using the QIAamp® MinElute™ Virus Spin Kit (Qiagen, Valencia, CA, USA), according to the manufacturer's protocol.

### HBV genotyping

Genotypes of HBV were determined using a line probe assay (INNO-LiPA HBV genotyping assay; Innogenetics NV, Ghent, Belgium) following manufacturer's recommendations and as previously described (Alzahrani et al., 2009).

### Precore/core gene amplification

Mutations in the preC/C region were amplified using a nested PCR protocol with the specific primers listed in Supplementary Table 1 and GoTaq® Green Master Mix (Promega, Madison, WI, USA). Nested primers were tagged with M13 sequences to enable direct sequencing after PCR amplification. For the first round of PCR, 5 μL of extracted DNA, and 12.5 pmol of each primer [HBVP+CoreF1 (+) and HBVP+CoreR1 (–)] were combined with GoTaq Green Master Mix in a total volume of 25 μL. The PCR reaction conditions were an initial denaturation step at 95°C for 3 min, followed by 35 amplification cycles (95°C for 1 min, 56°C for 45 s, and 72°C for 1 min), and a final extension step at 72°C for 7 min. For the second round of PCR, 2 μL of the first-round product was reamplified using the reaction mixture noted above, with the exception that HBVP+CoreF2 (+) and HBVP+CoreR2 (–) primers were used. The PCR conditions in this round were an initial denaturation step at 95°C for 3 min, followed by 30 amplification cycles (95°C for 1 min, 65°C for 45 s, and 72°C for 1 min), and a final extension step at 72°C for 7 min. Negative controls were amplified in parallel with samples. Ten microliters of amplified PCR products were separated on a 2% agarose gel, stained with ethidium bromide, then visualized under UV light. The PCR product of the expected size was excised from the gel and purified using a QIAquick Gel Extraction kit (Qiagen).

### DNA sequencing

Purified PCR products were directly sequenced using forward and reverse primers targeting the M13 sequence tags. Sequencing was performed on an ABI 3730 Genetic 16 Analyzer using a BigDye® Terminator v3.1 Cycle Sequencing kit (Applied Biosystems, Foster City, CA, USA) according to the manufacturer's protocol. Sequences from the 212-amino-acid preC/C region were assembled and edited using Lasergene software (DNASTAR, Madison, WI, USA), then aligned using the ClustalX multiple sequence alignment algorithm within the MegAlign module of the Lasergene software package (DNASTAR). Sequences were submitted to NCBI GenBank under accession numbers MH724953-MH725211.

### Statistical analyses

Statistical analyses were performed using Statistical Package for the Social Sciences (SPSS) software version 20.0 (SPSS Inc., Chicago, IL, USA). Age and ALT level, are expressed as the mean ± standard deviation. Chi square tests and Fisher's exact tests were used to compare categorical data. For all tests, *p*-values ≤ 0.05 were considered significant. Sliding window analysis was performed using DnaSP software version 5.10.1 [36] and GraphPad Prism software version 7.0 (GraphPad Software, La Jolla, California, USA). Sequence logos for the identified preC/C mutations were generated from HBV sequences derived from infected patients at various clinical stages using WebLogo software version 2.8.2 (http://weblogo.berkeley.edu; Crooks et al., 2004).

## Results

### General characteristics of clinical subjects

In total, 553 HBV-infected patients participated in the present study, and their baseline characteristics, including clinical features, viral load, and stage of HBV infection and liver disease are summarized in Table 1. All the patients participating in the present study were Saudi nationals. Among the HBV-infected patients, 339 were carriers of inactive HBV, 147 were carriers of active HBV, 45 were suffering from cirrhosis, and 22 had developed HCC. Advanced age was found to be a significant risk factor for HCC development (*p* < 0.0001); HCC patients had the highest mean age (60.23 ± 9.62 years), followed by cirrhosis patients (51.68 ± 10.84 years), while carriers of inactive (38.36 ± 12.18 years) and active HBV (35.12 ± 12.96 years) were of a similar age on average. Gender was also significantly associated with more severe liver disease (*p* = 0.005). Male patients predominated at all of the clinical stages of HBV infection, and this gender bias grew more pronounced with increasing disease severity; in the HCC patient group 95.5% of participants were male. Interestingly, BMI was significantly higher in active and inactive HBV carriers than in patients with advanced liver diseases (*p* = 0.018). While serum ALT levels were normal in carriers of inactive HBV, they were elevated in all other patient groups, although these differences were not statistically significant. Viral load was significantly elevated in the active HBV carrier, cirrhosis, and HCC patient groups compared with the inactive HBV carrier patients (*p* < 0.0001); the highest viral load was observed in the active HBV carrier group (5.13), followed by the HCC patient group (4.24), and the cirrhosis patient group (3.30).

**Table 1:** Baseline characteristics of all subjects included in the study.

**Variables**	**Inactive HBV** **carriers**	**Active HBV** **carriers**	**Liver** **Cirrhosis**	**Hepatocellular** **carcinoma**	***P*-value^a^**
	**(*n* = 339)**	**(*n* = 147)**	**(*n* = 45)**	**(*n* = 22)**	
Age (years)^*^	38.36 ± 12.18	35.12 ± 12.96	51.68 ± 10.84	60.23 ± 9.62	<**0.0001**
**Gender**°					
Male	229 (67.6%)	108 (73.5%)	38 (84.4%)	21 (95.5%)	**0.005**
Female	110(32.4%)	39 (26.5%)	7 (15.6%)	1 (4.5%)	
BMI^◇^	27.41 (24.84–31.13)	27.43 (23.11–31.56)	24.20 (21.58–30.37)	24.49 (21.48–27.77)	**0.018**
ALT^*^	27.74 ± 389.36	76.86 ± 86.78	75.71 ± 120.42	90.31 ± 87.37	0.895
Viral load_log10^◇^	2.66 (1.65–3.62)	5.13 (4.33–7.40)	3.30 (1.92–4.39)	4.24 (2.85–6.03)	<**0.0001**
**HBV Genotypes**°					
D [*n* = 494 (89.33%)]	302 (61.13%)	135 (27.33%)	37 (7.49%)	20 (4.05%)	0.257
E [*n* = 22 (3.98%)]	11 (50.00%)	5 (22.73%)	5 (22.73%)	1 (4.54%)	
Others [*n* = 37 (6.69%)]	26 (70.27%)	7 (18.92%)	3 (8.11%)	1 (2.70%)	

* Variables are expressed as Mean ± SD;

° Variables are expressed as count (%),

◇ *Variables are expressed as median interquartile range (25–75th). ^a^One way Anova and t-test for continuous data, chi square test for categorical data. Bold indicates significance*.

### HBV genotypes

The great majority of the subjects were infected with HBV genotype D (89.33%), followed by genotype E (3.98%). Other genotypes (6.69%) including HBV A, B, C, A/D and C/D were also detected (Table 1).

### The prevalence of HBV precore/core mutations in isolates from patients at different stages of clinical disease progression

We isolated HBV virus from all HBV-infected patients, then sequenced the preC/C region to look for mutations. Multiple alignment analysis of the preC/C region protein sequences against a reference sequence was then performed using BioEdit software, and sequence variations in HBV isolated from each of the clinical groups (IC, AC, LC, and HCC) were noted. Firstly, we identified the preC/C mutations that were associated with the progression of HBV-induced liver complications by combining data from the AC, LC, and HCC groups and comparing them with the mutation frequencies observed in the IC group (Table 2). Of 212 codons in this region (29 within the precore region and 183 within the core region), 34 contained mutations (Table 2). The prevalence of mutations at two loci was found to differ significantly between the IC group, exhibiting inactive HBV infection, and the combined AC+LC+HCC group of patients in whom the infection had progressed further. Mutation E77Q within the core region was significantly more prevalent in the AC+LC+HCC group than in the IC group (7.01 vs. 3.24% prevalence, respectively; *p* = 0.042). Similarly, mutation V91S was more prevalent in the combined group than in the IC group (3.27 vs. 0.59% prevalence, respectively; *p* = 0.031). Furthermore, mutation V91T, substituting valine for threonine at the same core position as V91S, was present in large percentage of patients in the AC+LC+HCC group; this prevalence (91.12%) was significantly higher than was seen in the IC group (9.44%; *p* < 0.0001).

**Table 2:** Prevalence of PreCore/Core mutations among inactive HBV carriers and active HBV carriers + cirrhosis + HCC.

**Mutations**	**Gene**	**IC (*n* = 339)**	**Percentage (%)**	**AC + LC + HCC(*n* = 214)**	**Percentage (%)**	***p*-value**
P5H	Precore	330	97.35	211	98.60	0.385
W28 stop codon	Precore	184	54.28	109	50.93	0.443
G29D	Precore	63	18.58	44	20.56	0.567
S12T	Core	231	68.14	153	71.50	0.404
S21A	Core	19	5.60	6	2.80	0.123
I27V	Core	308	90.86	193	90.19	0.793
S35A	Core	7	2.06	6	2.80	0.576
A34T	Core	9	2.65	3	1.40	0.385
Y38F	Core	30	8.85	13	6.07	0.235
E40D	Core	71	20.94	40	18.69	0.52
E64D	Core	99	29.20	65	30.37	0.769
M66I	Core	20	5.90	13	6.07	0.933
N67AS	Core	25	7.37	20	9.35	0.409
N67T	Core	266	78.47	161	75.23	0.377
A69S	Core	21	6.19	12	5.61	0.776
S74G	Core	304	89.68	191	89.25	0.874
E77D	Core	8	2.36	6	2.80	0.746
E77Q	Core	11	3.24	15	7.01	**0.042**
A80I	Core	133	39.23	88	41.12	0.659
A80T	Core	133	39.23	76	35.51	0.38
A80V	Core	32	9.44	27	12.62	0.238
E83D	Core	322	94.99	205	95.79	0.662
S87G	Core	11	3.24	7	3.27	0.987
V91S	Core	2	0.59	7	3.27	**0.031**
V91T	Core	32	9.44	195	91.12	<**0.0001**
M93V	Core	25	7.37	16	7.48	0.964
I97F	Core	327	96.46	208	97.20	0.635
E113Q	Core	12	3.54	6	2.80	0.635
L116I	Core	244	71.98	162	75.70	0.334
P130A	Core	1	0.29	4	1.87	0.076
P130Q	Core	20	5.90	14	6.54	0.759
A131P	Core	22	6.49	16	7.48	0.655
T147A	Core	17	5.01	10	4.67	0.856
V149I	Core	63	18.58	32	14.95	0.27
S155T	Core	48	14.16	28	13.08	0.721
Q177K	Core	19	5.60	11	5.14	0.814
R179P	Core	14	4.13	9	4.21	0.965
E180G	Core	17	5.01	11	5.14	0.948
S181P	Core	46	13.57	34	15.89	0.45
184 stop codon	Core	325	95.87	203	94.86	0.578

*Bold indicates significance*.

**Figure 1:**
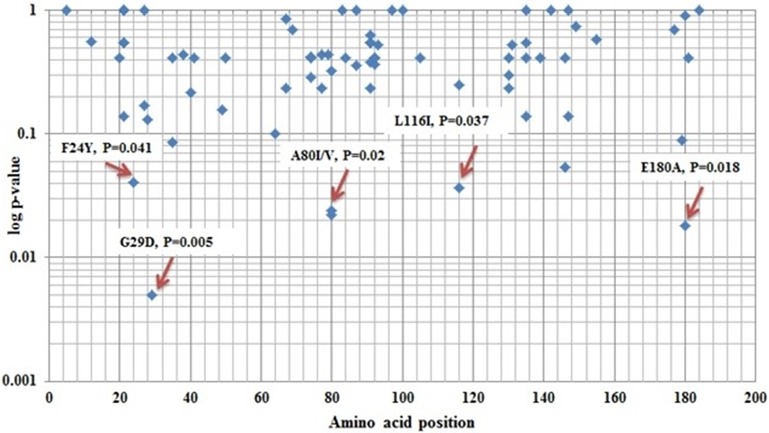
Different amino acid position variations between Active HBV carrier's vs. Liver Cirrhosis of core protein.

Next, we identified mutations that may be linked to the development of cirrhosis in HBV-infected patients by comparing the frequency of preC/C mutations in active HBV carriers with those in the LC group. The amino acid positions of all of the observed mutations, and *p*-values denoting the significance of differential prevalence between the two groups, are shown in Figure 1. In total, four mutations were significantly more prevalent in the LC group than in the AC group; mutation G29D in the precore region (31.11 and 12.93% prevalence in the LC and AC groups, respectively; *p* = 0.005), mutation F24Y in the core region (6.67 and 0.68% prevalence in the LC and AC groups, respectively; *p* = 0.041), mutation A80V in the core region (22.22 and 9.52% prevalence in the LC and AC groups, respectively; *p* = 0.024), and mutation E180A (11.11 and 2.04% prevalence in the LC and AC groups, respectively; *p* = 0.018). Conversely, however, two core mutations were significantly more prevalent in the AC group than in the LC group; mutation A80I (48.30 and 28.89% prevalence in the AC and LC groups, respectively; *p* = 0.022), and mutation L116I (79.59 and 64.44% prevalence in the AC and LC groups, respectively; *p* = 0.037).

To identify mutations that may be indicators of severe liver disease, the prevalence of preC/C mutations in the AC group was compared with those in the combined LC and HCC groups (LC+HCC). The comparative analysis of the prevalence of these mutations within the two groups is shown in Table 3. There were eight point mutations whose prevalence differed significantly between the AC and LC+HCC groups, including the precore mutation W28^*^ (tryptophan to stop) which has been reported previously, and is known to be involved in liver disease progression (64.18 and 44.90% prevalence in the LC+HCC and AC groups, respectively; *p* = 0.009). The majority of mutations with differential prevalence between the groups were more common in the LC+HCC group. These included the precore mutation G29D (37.31 and 12.93% prevalence in the LC+HCC and AC groups, respectively; *p* < 0.0001), the core mutation E64D (41.79 and 25.17% prevalence in the LC+HCC and AC groups, respectively; *p* = 0.014), the core mutation E77Q (13.43 and 4.08% prevalence in the LC+HCC and AC groups, respectively; *p* = 0.02), the core mutation A80T (47.76 and 29.93% prevalence in the LC+HCC and AC groups, respectively; *p* = 0.011) and the core mutation A80V (19.40 and 9.52% prevalence in the LC+HCC and AC groups, respectively; *p* = 0.044). Conversely, two point mutations in the core region were present at significantly higher frequency in the AC group than in the LC+HCC group; mutation A80I (48.30 and 25.37% prevalence in the AC and LC+HCC groups, respectively; *p* = 0.002), and mutation L116I (79.59 vs. 67.16% prevalence in the AC and LC+HCC groups, respectively; *p* = 0.049). It is interesting to note that mutations at a given position may be more or less prevalent at the later stages of liver disease, depending on the exact substitution made.

**Table 3:** Prevalence of preCore/Core mutations among active HBV carriers and cirrhosis+HCC.

	**Gene**	**AC (*n* = 147)**	**Percentage (%)**	**LC + HCC (*n* = 67)**	**Percentage (%)**	***p*-value**
P5H	Pre core	145	98.64	66	98.51	1
W28 stop codon	Pre core	66	44.90	43	64.18	**0.009**
G29D	Pre core	19	12.93	25	37.31	<**0.0001**
S12T	Core	111	75.51	42	62.69	0.054
S21A	Core	4	2.72	2	2.99	1
I27V	Core	129	87.76	64	95.52	0.077
S35A	Core	2	1.36	4	5.97	0.078
Y38F	Core	6	4.08	7	10.45	0.118
E40D	Core	24	16.33	16	23.88	0.189
E64D	Core	37	25.17	28	41.79	**0.014**
M66I	Core	7	4.76	6	8.96	0.234
N67AS	Core	11	7.48	9	13.43	0.166
N67T	Core	113	76.87	48	71.64	0.411
A69S	Core	7	4.76	5	7.46	0.523
S74G	Core	132	89.80	59	88.06	0.704
E77D	Core	2	1.36	4	5.97	0.078
E77Q	Core	6	4.08	9	13.43	**0.02**
A80I	Core	71	48.30	17	25.37	**0.002**
A80T	Core	44	29.93	32	47.76	**0.011**
A80V	Core	14	9.52	13	19.40	**0.044**
E83D	Core	140	95.24	65	97.01	0.723
S87G	Core	5	3.40	2	2.99	1
V91S	Core	4	2.72	3	4.48	0.68
V91T	Core	135	91.84	60	89.55	0.586
M93V	Core	12	8.16	4	5.97	0.572
I97F	Core	142	96.60	66	98.51	0.668
L116I	Core	117	79.59	45	67.16	**0.049**
P130A	Core	1	0.68	3	4.48	0.092
P130Q	Core	11	7.48	3	4.48	0.556
A131P	Core	13	8.84	3	4.48	0.26
T147A	Core	7	4.76	3	4.48	1
V149I	Core	23	15.65	9	13.43	0.674
S155T	Core	21	14.29	7	10.45	0.44
Q177K	Core	7	4.76	4	5.97	0.743
R179P	Core	4	2.72	5	7.46	0.142
E180G	Core	6	4.08	5	7.46	0.326
S181P	Core	19	12.93	15	22.39	0.079
184 stop codon	Core	138	93.88	65	97.01	0.509

*Bold indicates significance*.

### The association between precore/core region mutations and biochemical factors and disease progression

Next, we performed univariate and multivariate logistic regression analyses to investigate associations between preC/C region mutations and clinical characteristics, and disease progression. Univariate analyses revealed that gender (*p* = 0.008), HBV viral load (*p* < 0.0001), and core mutation E77Q (*p* = 0.047) all displayed a significant positive correlation with disease progression when the IC group was compared with the AC+LC+HCC combined group (Table 4). Furthermore, multivariate analysis revealed a significant association between disease progression and both patient age (*p* < 0.0001) and HBV viral load (*p* < 0.0001), when the IC group was compared with the AC+LC+HCC patient group (Table 4). A80V mutation also showed significant association in this analysis with a *p*-value of 0.05 (Table 4).

**Table 4:** Univariate and multivariate logistic analysis among inactive HBV carriers vs. active HBV carriers + cirrhosis + HCC.

**Variables**	**Univariate analysis**				**Multivariate analysis**			
	**Odds ratio**	**95% C.I**.		***P*-value**	**Odds ratio**	**95% C.I**.		***P*-value**
		**Lower**	**Upper**			**Lower**	**Upper**	
Age	1.01	0.998	1.023	0.101	1.054	1.031	1.077	<**0.0001**
Gender	0.587	0.395	0.872	**0.008**	1.027	0.573	1.841	0.929
ALT	1.000	1.000	1.001	0.476	1.00	0.999	1.001	0.968
BMI	0.969	0.936	1.002	0.069	0.96	0.92	1.003	0.066
Viral Load	1.363	1.258	1.476	<**0.0001**	1.733	1.505	1.995	<**0.0001**
W28 Stop codon	1.144	0.812	1.611	0.443	0.938	0.472	1.866	0.856
G29D	0.882	0.574	1.356	0.567	1.016	0.466	2.214	0.969
F24Y	1.927	0.613	6.053	0.262	1.423	0.328	6.168	0.637
E64D	0.946	0.651	1.374	0.769	1.648	0.864	3.146	0.130
E77Q	0.445	0.2	0.988	**0.047**	0.54	0.154	1.892	0.336
A80I	0.948	0.671	1.340	0.763	0.828	0.28	2.453	0.733
A80V	0.722	0.419	1.243	0.24	0.272	0.074	0.998	**0.050**
A80T	1.172	0.822	1.672	0.38	1.031	0.337	3.158	0.957
V91T	0.909	0.498	1.659	0.756	0.923	0.383	2.219	0.857
L116I	0.852	0.577	1.257	0.419	0.581	0.299	1.129	0.109
E180A	0.945	0.38	2.351	0.903	1.415	0.241	8.295	0.700
Genotype D	0.935	0.535	1.634	0.814	0.865	0.145	5.159	0.873
Genotype E	1.107	0.32	3.827	0.872	0.479	0.053	4.322	0.512
Other genotypes	1.057	0.574	1.948	0.858	1.372	0.215	8.745	0.738

*Bold indicates significance*.

We then performed similar univariate analyses of the association between clinical features and HBV mutations and disease progression, this time considering the progression from the AC to the LC groups. These analyses revealed significant associations between HBV-related cirrhosis and age (*p* < 0.0001), viral load (*p* = 0.001), precore mutation G29D (*p* = 0.006), core mutation F24Y (*p* = 0.045), core mutation A80I (*p* = 0.024), core mutation L116I (*p* = 0.04), and core mutation E180A (*p* = 0.017; Table 5). Multivariate analysis between the AC and LC groups indicated that age (*p* = 0.001), gender (*p* = 0.018), HBV viral load (*p* = 0.012) and core mutation L116I (*p* = 0.021) were significantly correlated with HBV-related cirrhosis.

**Table 5:** Univariate and multivariate logistic analysis among active HBV carriers vs. cirrhosis group.

**Variables**	**Univariate analysis**				**Multivariate analysis**			
	**Odds ratio**	**95% C.I**.		***P*-value**	**Odds ratio**	**95% C.I**.		***P*-value**
		**Lower**	**Upper**			**Lower**	**Upper**	
Age	1.117	1.077	1.158	<**0.0001**	1.191	1.070	1.326	**0.001**
Gender	0.524	0.216	1.272	0.153	0.041	0.003	0.584	**0.018**
ALT	0.999	0.995	1.003	0.595	1.003	0.994	1.012	0.556
BMI	0.941	0.878	1.009	**0.088**	0.884	0.758	1.031	0.115
Viral Load	0.808	0.713	0.914	**0.001**	0.452	0.243	0.840	**0.012**
W28 Stop codon	0.595	0.303	1.169	0.132	7.161	0.420	122.21	0.174
G29D	0.329	0.149	0.727	**0.006**	0.271	0.008	9.068	0.466
F24Y	0.096	0.01	0.946	**0.045**				0.999
E64D	0.554	0.273	1.125	0.102	0.256	0.024	2.704	0.257
E77Q	0.596	0.143	2.485	0.477	1.695	0.007	389.432	0.849
A80I	2.300	1.118	4.730	**0.024**				0.999
A80V	0.461	0.200	1.064	**0.07**				0.999
A80T	0.704	0.35	1.415	0.324				0.999
V91T	0.895	0.292	2.746	0.846	5.612	0.343	91.965	0.227
L116I	2.152	1.037	4.467	**0.040**	18.943	1.553	231.03	**0.021**
E180A	0.167	0.038	0.727	**0.017**				0.999
Genotype D	2.432	0.926	6.389	0.071				0.998
Genotype E	0.297	0.041	2.168	0.231				0.998
Other genotypes	0.474	0.162	1.387	0.173				0.998

*Bold indicates significance*.

Several statistically significant associations were also observed in similar analyses when the AC group was compared with LC+HCC group. Univariate analysis showed that age (*p* < 0.0001), gender (*p* = 0.022), BMI (*p* = 0.023), HBV viral load (*p* < 0.0001), precore mutation W28^*^ (*p* = 0.01), precore mutation G29D (*p* < 0.0001), core mutation E64D (*p* = 0.015), core mutation E77Q (*p* = 0.019), core mutation A80I (*p* = 0.002), core mutation A80T (*p* = 0.012), and core mutation L116I (with a border line *p*-value of 0.051) were all significantly correlated with HBV-related LC+HCC (Table 6). Furthermore, multivariate regression analysis revealed that age (*p* < 0.0001), gender (*p* = 0.020), HBV viral load (*p* = 0.014), and core mutation L116I (*p* = 0.026) were independently associated with cirrhosis HCC in HBV-infected patients (Table 6).

**Table 6:** Univariate and multivariate logistic analysis among active HBV carriers vs. cirrhosis + HCC combined group.

**Variables**	**Univariate analysis**				**Multivariate analysis**			
	**Odds ratio**	**95% C.I**.		***P*-value**	**Odds ratio**	**95% C.I**.		***P*-value**
		**Lower**	**Upper**			**Lower**	**Upper**	
Age	1.137	1.097	1.178	<**0.0001**	1.168	1.087	1.256	<**0.0001**
Gender	0.382	0.167	0.872	**0.022**	0.079	0.009	0.671	**0.020**
ALT	0.999	0.996	1.002	0.599	1.001	0.994	1.009	0.746
BMI	0.93	0.873	0.990	**0.023**	0.907	0.806	1.020	0.105
Viral Load	0.795	0.713	0.887	<**0.0001**	0.586	0.830	0.897	**0.014**
W28 Stop codon	0.455	0.251	0.825	**0.010**	5.822	0.616	54.989	0.128
G29D	0.249	0.125	0.498	<**0.0001**	0.288	0.036	2.304	0.241
F24Y	0.146	0.015	1.432	0.099				0.999
E64D	0.469	0.254	0.864	**0.015**	0.289	0.044	1.880	0.194
E77Q	0.274	0.093	0.805	**0.019**	0.636	0.053	7.602	0.721
A80I	2.748	1.451	5.202	**0.002**				0.999
A80V	0.534	0.247	1.152	0.110				0.999
A80T	0.467	0.258	0.847	**0.012**				0.999
V91T	0.893	0.33	2.415	0.824	3.178	0.233	43.279	0.385
L116I	1.907	0.997	3.648	0.051	7.679	1.270	46.432	**0.026**
E180A	0.258	0.06	1.115	0.070				0.999
Genotype D	1.974	0.807	4.828	0.136				0.999
Genotype E	0.448	0.062	3.252	0.427				0.999
Other genotypes	0.538	0.202	1.432	0.215				0.999

*Bold indicates significance*.

### Analysis of amino acid diversity in the precore/core region

**Figure 2:**
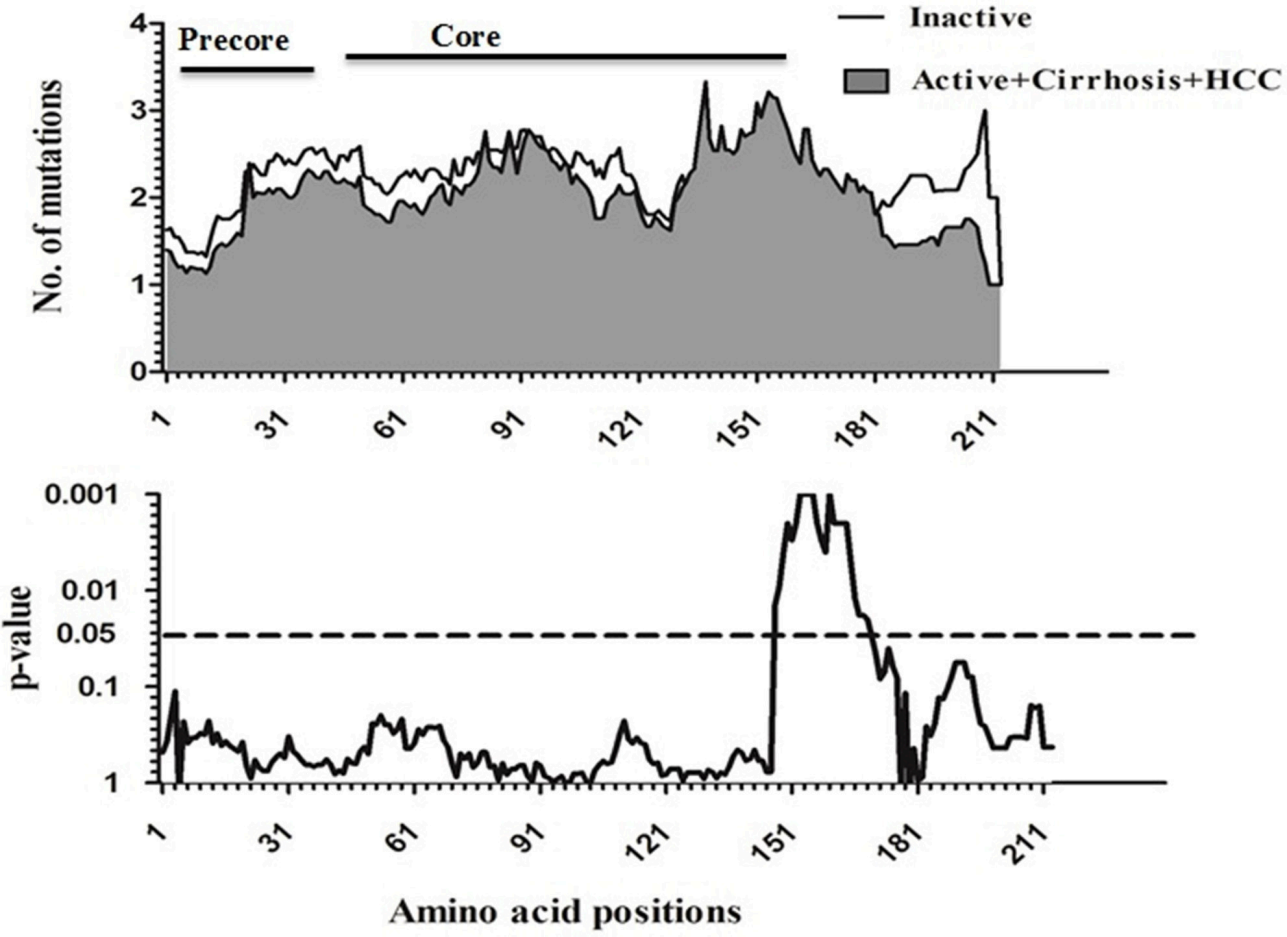
Sliding-window analysis for the differences in amino acid changes between the Inactive and active+ cirrhosis+ HCC groups in HBV infected patients. The first panel shows the mean number of amino acid mutations of inactive (*thick line*) and active+ cirrhosis+HCC (*shaded area*) in each sliding window of30 amino acids. Second panels show the probability of observed differences in the amino acid mutations between inactive and active+ cirrhosis+ HCC was calculated for each window by *t*-test and was plotted for an inverted logarithmic scale. Significant differences observed in amino acid changes from 147 to 176.

**Figure 3:**
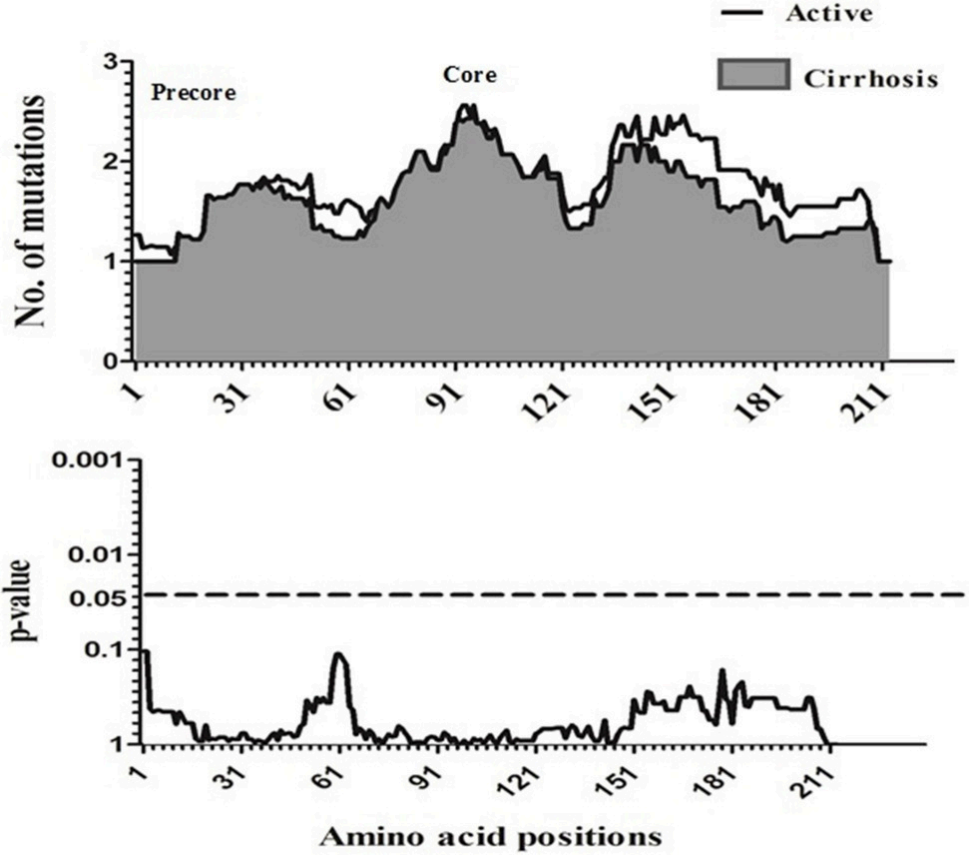
Sliding-window analysis for the differences in amino acid changes between the active and liver cirrhosis patients. The ftrst panel shows the mean number of amino acid mutations of active (*thick line*) and cirrhosis (*shaded area*) in each sliding window of 30 amino acids. Second panels show the probability of observed differences in the amino acid mutations between active and cirrhosis was calculated for each window by *t*-test and was plotted for an inverted logarithmic scale. No Significant differences observed in amino acid.

**Figure 4:**
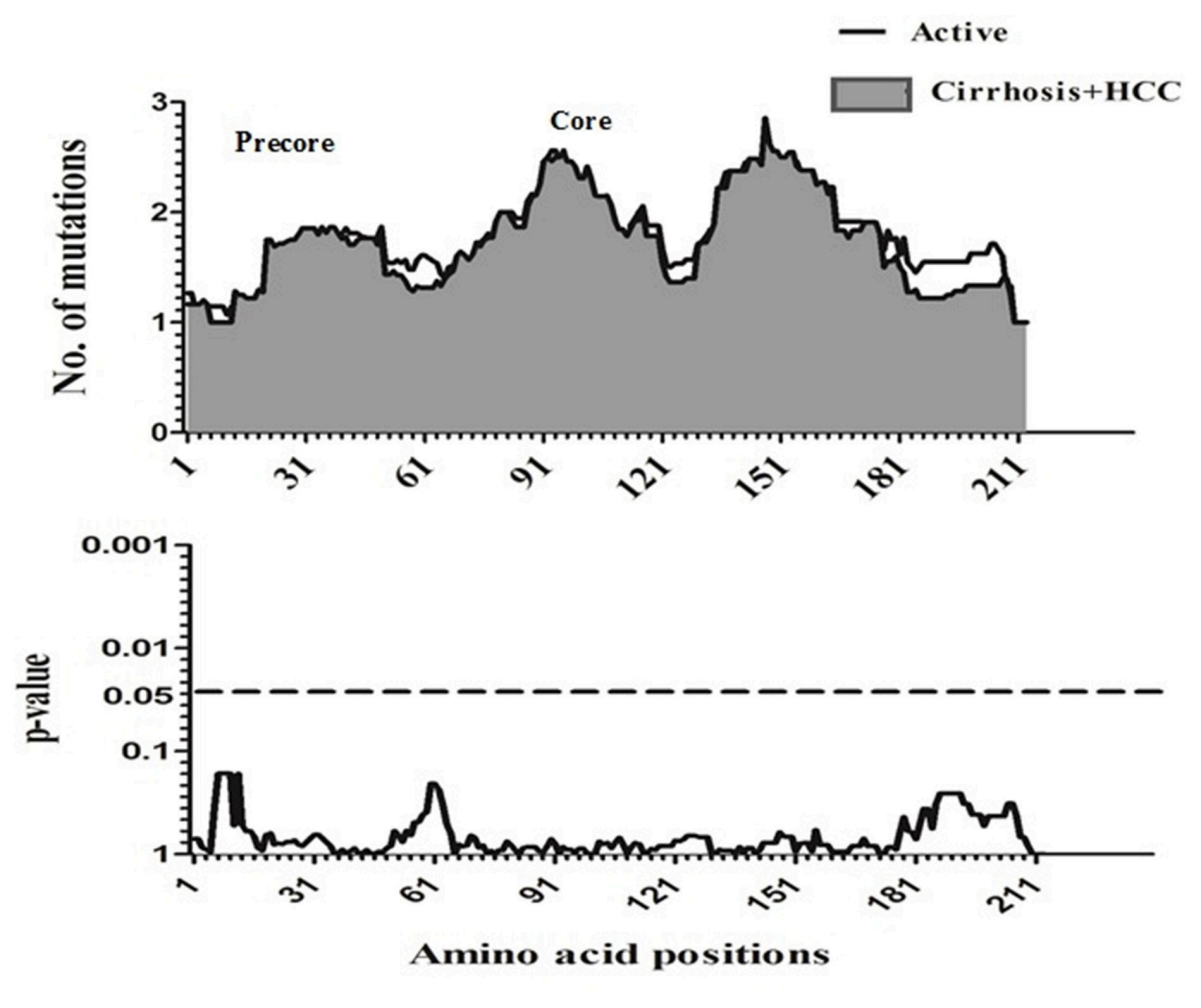
Sliding-window analysis for the differences in amino acid changes between the active and liver cirrhosis + HCC patients. The ftrst panel shows the mean number of amino acid mutations of active (*thick line*) and cirrhosis + HCC (*shaded area*) in each sliding window of 30 amino acids. Second panels show the probability of observed differences in the amino acid mutations between active and cirrhosis + HCC was calculated for each window by *t*-test and was plotted for an inverted logarithmic scale. No significant differences observed in amino acid.

To compare amino acid diversity across the entire preC/C region in the different HBV-infected patient groups, a sliding window analysis was performed. When the IC group was compared with the combined group representing more severe disease (AC+LC+HCC), a statistically significant difference in the number of mutations was observed in the region between amino acid positions 147 and 176 (corresponding to codons 118 to 147 of the core region; *p* = 0.002) (Figure 2). Conversely, no significant variation in amino acid diversity was detected when the AC group was compared to either the LC group (Figure 3) or the combined LC+HCC group (Figure 4).

### Determination of sequence logos significant amino acid mutations in the core region

**Figure 5:**
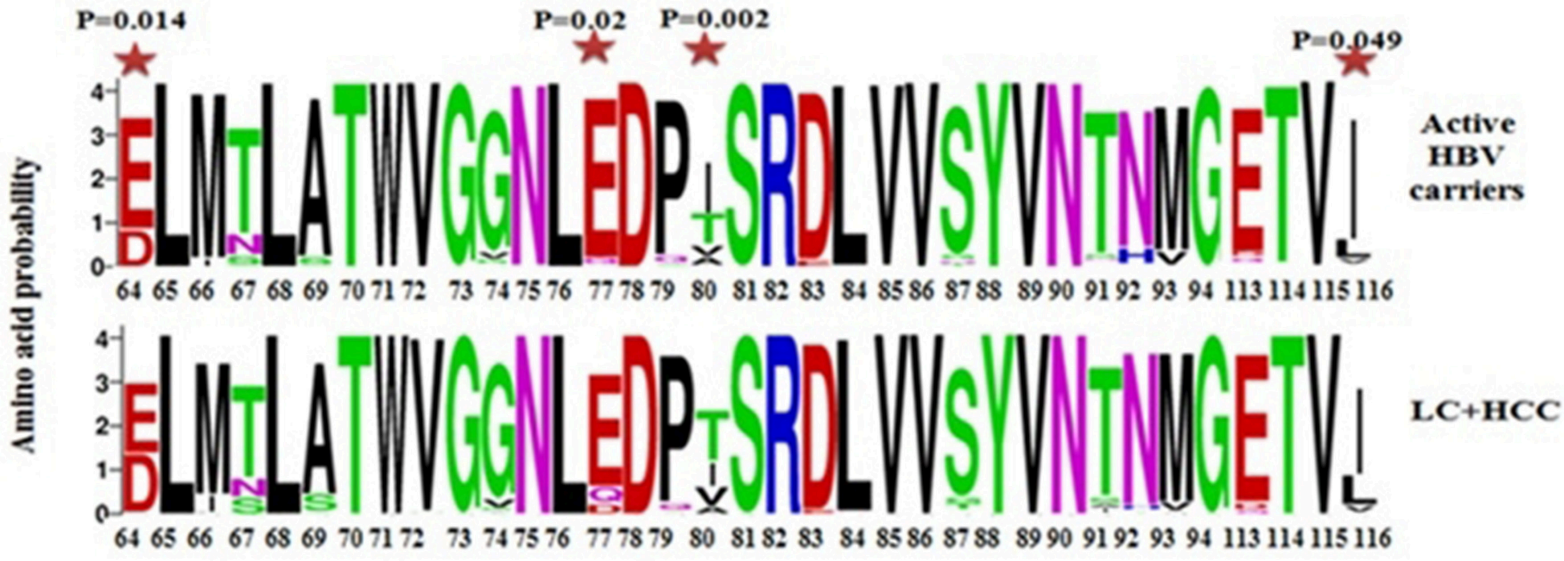
Sequence logos depicting the variations in frequency of identified mutations at different positions of the core protein between the active HBV carriers and HBV -related liver cirrhosis + hepatocellular carcinoma patients. 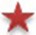 Statistically significant differences between active HBV carriers vs. liver cirrhosis + HCC.

Next, we compared the relative amino acid prevalence at mutational hotspots between the AC and LC+HCC groups, and depicted these variations in the form of mutation sequence logos (Figure 5). The amino acids at the four positions in the core protein that were significantly associated with disease progression (E64D, E77D/Q, A80I/T/V, and L116I) were found to be more variable in the LC+HCC group than in the AC group. Considering the core mutation E77D/Q, D is absent in AC group but detectable at low frequency in the LC+HCC group. With the core mutation A80I/T/V, I is the most frequent amino acid in the AC group, followed by T and then V, whereas in the LC+HCC group T is the most frequent, followed by I and then V. Similarly, at position 116, L is present at higher frequency in the LC+HCC group than in the AC group (Figure 5).

## Discussion

HBV infection and the subsequent development of severe liver disease continues to be a major health concern, both in Saudi Arabia and globally, despite the existence of an efficient vaccination program and antiviral medications. Patients infected with HBV exhibit a different spectrum of diseases characterized by varying degree of liver damage. Therefore, it is of utmost importance to determine the severity of liver impairment before the start of any type of treatment. Elevated serum ALT level has universally been accepted as an independent criterion (asides from HBV DNA levels) for institution of treatment. All international and local guidelines (Abaalkhail et al., 2014; Terrault et al., 2016) recommend utilizing measurement of ALT for the ascertainment of treatment need. This has been based on many studies that have shown elevated ALT as a marker of significant hepatic fibrosis and progression to cirrhosis (Kumar et al., 2008; Sanai et al., 2011). As such IC has been defined as those with low HBV DNA and normal ALT levels (Abaalkhail et al., 2014; Terrault et al., 2016). These patients have the lowest likelihood of developing significant fibrosis, and hence cirrhosis (Kumar et al., 2008; Sanai et al., 2011; Abaalkhail et al., 2014; Terrault et al., 2016). As such this is the basis for instituting antiviral treatment for AC, and excluding those who are IC who constitute the most benign form of the disease.

Previously, mutations in the surface, X gene, and precore/core regions of HBV have been associated with the severity and progression of infection (Datta et al., 2014; Zhang et al., 2016). The precore and core regions of the HBV genome encode the HBeAg and HBcAg proteins, respectively; while these two proteins contain different antigenic epitopes, both are known to be important in the modulation of HBV pathogenesis (Alexopoulou et al., 2009). Some precore/core gene mutations are predicted to cause a conformational change in the corresponding protein, resulting in an increased ability to escape immune surveillance and a greater chance of developing severe liver disease (Mohamadkhani et al., 2009). The present study aimed to identify and investigate amino acid changes in the complete precore/core region at different clinical stages of HBV infection. One of the most common precore mutations, detected in more than 50% of individuals with chronic hepatitis B in Asia and the Mediterranean area, is a G to A substitution at nucleotide 1896, resulting in a tryptophan to stop codon substitution at codon 28 (Tong et al., 2005). This mutation is known to abolish HBeAg production, thus affecting HBV replication and liver disease progression (Carman et al., 1989; Alexopoulou, 2009; Kim et al., 2016; Zhang et al., 2016). In the present study, we observed a statistically significant increase in preC-W28^*^ frequency in the LC+HCC group of patients as compared with active HBV carriers, which is consistent with previous reports of a positive association between this mutation and liver disease severity (Kim et al., 2012; Park et al., 2014; Xie et al., 2015). Another precore mutation, which is usually detected alongside preC-W28^*^, is G29D; a recent meta-analysis combined the results of eleven individual studies and confirmed a significant correlation between G29D mutation and an increased risk of HCC (Liao et al., 2012). Similarly, the present analysis identified a significant positive association between the mutation G29D and progression toward severe liver disease in HBV-infected Saudi patients.

Previously, HBcAg was identified as an important target for immune-mediated viral clearance, achieved primarily through the induction of B-cell, T helper cell, and cytotoxic T lymphocyte (CTL) responses (Chisari, 2000; Pumpens and Grens, 2001). Several key immune recognition sites along the HBcAg protein have been noted, including target epitopes for human CD4^+^ T-cells (amino acids 1–20, 50–69, 81–105, 117–131, and 141–165), cytotoxic T lymphocyte/CD8^+^ T-cells (amino acids 18–27, 88–96, 130–140, and 141–151), and B-cells (amino acids 74–89, 107–118, and 127–138) (Alexopoulou, 2009; Kim et al., 2012; Zhu et al., 2012). Mutations in such immuno-active regions of HBcAg are vital for viral persistence, host immune responses, and the direction of progress of HBV infection (Bozkaya et al., 1996; Pumpens et al., 2002; Alexopoulou et al., 2009). Consistent with previous reports (Kim et al., 2012; Zhu et al., 2012), a non-random distribution of mutations was observed in the immuno-active and immuno-inactive regions of the core protein in this study. The location of mutations that are associated with HBV-related disease progression are variable; Kim et al. (2012) found that such core protein mutations were primarily located within CD4^+^ T-cell epitopes that were designated MHC class II restricted regions (Kim et al., 2012), while Carman et al. (1997) found that HBcAg mutations associated with progressive disease were concentrated in B-cell epitopes (Carman et al., 1997). A separate study of Indian patients infected with HBV genotype D identified advantageous mutations in B-cell epitopes during the progression of chronic hepatitis B infection (Mondal et al., 2015). Similarly, Assar et al. (2012) reported several mutations responsible for immune evasion in T helper and B-cell epitopes in Iranian patients infected with HBV genotype D (Assar et al., 2012). In the present study, three significant mutations were located within T-cell epitopes (F24Y, E64D, and V91S/T), and an equal number of significant mutations were present within B-cell epitopes (E77Q, A80I/V, and L116I), of the HBcAg protein. Viral genomic mutation is a primary strategy for immune evasion, and during the course of chronic HBV infection there is increasing core protein variation. Indeed, several emerging *de novo* core mutations are known to alter core antigenicity (Alexopoulou, 2009). Furthermore, mutations in the major HBcAg T-cell epitopes have been proposed to facilitate immune escape, resulting in disease-free HBV persistence, whereas mutations within B-cell epitopes may result in an ineffective antibody response, leading to disease progression and selection for specific B-cell epitope mutants (Carman et al., 1997; Zhu et al., 2012). In the present study, the HBcAg mutations that were associated with advanced liver disease were derived from all three epitope types (B-cell, CD4^+^ T-cell, and CD8^+^ T-cell), suggesting a complex interplay between the virus and the host throughout the course of chronic HBV infection. Mutation E180A, which was significantly associated with liver disease development, was not located in a known epitopic region, suggesting that mechanisms other than immune evasion may be involved. However, it is important that these observations are verified by further detailed experiments. Of importance, we have performed extensive structural modeling of these mutations, however, none of the mutations showed any significant changes in the overall 3-dimensional structure of the core protein.

In the present study, two statistically significant core protein amino acid substitutions (E77Q and V91S/T) that may influence the progression of HBV infection from an inactive carrier stage to more advanced stages were identified. Similarly, four core protein mutations that were associated with the progression from active HBV status to cirrhosis were noted (F24Y, A80I/V, L116I, and E180A). Furthermore, progression from active HBV status to cirrhosis and HCC was associated with four core protein substitutions: E64D, E77Q, A80I/T/V, and L116I. Some of these mutations, such as E77Q, A80I/V, and L116I, are present at multiple stages of HBV infection, whereas others are associated with a specific clinical stage. There are many mechanisms that can potentially explain these observations, including the presence of a diverse population of core variants at different stages throughout chronic HBV infection, as suggested by Alexopoulou (2009).

The precise mutations that are identified differ between studies, suggesting that HBV genotype and population variation play significant roles. Interestingly, mutation E77Q, detected in our study, was also reported by Tong et al. (2005) to be within the B-cell epitope and to abolish core protein and HBeAg recognition by a rabbit polyclonal antibody; this mutation may therefore confer the ability to evade anti-HBc or anti-HBe immune attack (Tong et al., 2005). Core mutation V91S/T was significantly associated with disease progression in HBV-infected patients, which is consistent with previous reports of an amino acid hot spot between residues 81–105 that is important for the progression of disease in chronically infected patients (Kim et al., 2012). Very few studies comparing HBV core mutations in active HBV carriers and cirrhotic patients have been reported to date. Mohamadkhani et al. reported an association between CTL epitope HBV mutations and liver fibrosis (Mohamadkhani et al., 2009), and Mohebbi et al. (2012) identified several mutations present in immuno-active HBV core protein regions that occurred with differing frequency during chronic HBV infection and liver cirrhosis. We identified four core protein mutations that were significantly associated with cirrhosis: F24Y, A80I/V, L116I, and E180A. Codon 24 is within the CTL epitope (amino acids 18–27), which is exposed on the outer surface of core protein, and mutation at this position is associated with HBV persistence (Sendi et al., 2009; Mina et al., 2015). Several studies have linked mutations in core protein amino acids 21–34 with a severe exacerbation of chronic hepatitis B infection (Ehata et al., 1994), and core protein mutations have also been associated with HCC (Ni et al., 2003; Kim et al., 2012).

In this study, the HBcAg mutations E64D, E77Q, A80I/T/V, and L116I were significantly associated with cirrhosis and HCC. Previously, a study of HBV core variability under antiviral therapy reported the presence of the E64D mutation during the course of HBV infection, and stated that this substitution significantly reduced T-cell proliferation *in vitro* when it occurred with mutation T67N (Homs et al., 2011). Similarly, the mutations associated with severe liver disease were reportedly concentrated in the center of HBcAg, in a region including amino acids 59–66 (Akarca and Lok, 1995). Several studies have observed that the core protein region encompassing amino acids 80–120 is mutated more frequently, leading to viral persistence and a higher risk of developing severe liver disease, including HCC (Sendi et al., 2009; Kim et al., 2012; Zhu et al., 2012; Mondal et al., 2015). In contrast, we observed the mutations A80I/V and L116I more frequently in active HBV carriers than in patients with severe liver disease. Indeed, both A80I/V and L116I were significantly associated with a decreased risk of cirrhosis and HCC in HBV-infected patients. There have been other reports of mutations within or flanking the epitopes within the HBcAg protein reducing the risk of severe liver disease in HBV-infected patients (Sung et al., 2009). One possible explanation for such a protective effect against HCC is that some mutants could cause reduction in viral load (Sung et al., 2009). Multivariate logistic regression analysis indicated that the A80V and L116I mutations may also be useful independent prognostic markers of HBV-associated liver disease progression, particularly in combination with other contributing factors, such as viral load. This finding could result in better clinical management of HBV-infected patients, before the development of severe liver complications. Previously identified risk factors for severe liver disease in chronic hepatitis B patients include male sex, old age, high serum ALT levels, HBeAg mutation, and high viral load (Datta et al., 2014). Consistent with this, in HBV-infected Saudi patients we identified advanced age, male sex, BMI, and high viral load as significant predictors of the progression to advanced liver disease. However, no significant association was found when we used HBV genotype as a potential confounding factor in the multivariate analysis.

This study has a few limitations, one of which is due to the inherent difficulty of characterizing chronic HBV infection, and another is from the usual imperfections of a cross-sectional design. Clearly, the study does not represent a longitudinal analysis of a prospectively followed cohort. It should be noted that the preC/C mutations were only tested at a single time-point along the course of the infection. This raises the possibility that the identified mutations in those with HBV-related liver complications may not be preexisting mutations in those at risk, but could have arisen after the development of these complications. To test this hypothesis further, it would require the prospective follow up of non-complicated HBV patients with existing mutations, to evaluate for the future development of cirrhosis and HCC. This, obviously, is beyond the scope of the current study, and could be validated in future, longitudinally followed, prospective cohorts.

In conclusion, we have shown that mutations in the preC/C region of the HBV genome play an important role in determining the severity of HBV-associated liver complications. To date, there have been a limited number of studies exploring HBV genetic variation within infected Saudi patients, and this study greatly improves our understanding of HBV preC/C mutations, particularly within antigenic epitopes. Prospective studies exploring the functional mechanisms of the mutations identified in this study will have long-term implications for the treatment of HBV-infected patients within the Saudi population.

## Author contributions

AA-Q, HA-A, MNA-A, AAA, and FS conceived and designed the experiments. MRA-A, DD, and MB performed the experiments. MRA-A, AAA, AA, WA-H, KA, MK, SKA, and AE-S analyzed the data. AAA, FS, HA-A, MK, WA-H, KA, and MK contributed reagents, materials, analysis tools. AA-Q, NN, and MNA-A wrote the paper.

### Conflict of interest statement

The authors declare that the research was conducted in the absence of any commercial or financial relationships that could be construed as a potential conflict of interest.
