# Supplementary material for: The Correlation Between Hepatitis B Virus Precore/Core Mutations and the Progression of Severe Liver Disease
Source: Front Cell Infect Microbiol. 2018 Oct 22;8:355. doi: 10.3389/fcimb.2018.00355 (PMC6204459; doi:10.3389/fcimb.2018.00355)
Supplement: Supplementary file 1 [file Table_1.DOCX]

| **Primers** | **Position** | **Sequence** | **size** |
| --- | --- | --- | --- |
| **Round 1** |  |  |  |
| HBVP+CoreF1 (+) | 1632-1653 | 5’ CAG GTC TTG CCC AAC GTC TTA 3’ | 976 bp |
| HBVP+CoreR1 (-) | 2587- 2608 | 5’ CTG TCA GAG GGC CCA CAT ATT 3’ |  |
| **Round 2** |  |  |  |
| HBVP+CoreF2 (+) | 1688-1709 | 5’ GAC CGA CCT TGA GGC ATA TTT 3’ | 791 bp |
| HBVP+CoreR2 (-) | 2458-2479 | 5’ TCC CAC CTT ATG AGT CCA AGG 3’ |  |

Supplementary table 1: Primers used to amplify the PreCore/Core region

.
